# Health Information Needs of Young Chinese People Based on an Online Health Community: Topic and Statistical Analysis

**DOI:** 10.2196/30356

**Published:** 2021-11-08

**Authors:** Jie Wang, Xin Wang, Lei Wang, Yan Peng

**Affiliations:** 1 School of Management Capital Normal University Beijing China; 2 State key Laboratory of Networking and Switching Technology Beijing University of Posts and Telecommunications Beijing China; 3 Department of Electrical and Computer Engineering The State University of New York at Stony Brook Stony Brook, NY United States

**Keywords:** information needs, young people, online health community, topic analysis

## Abstract

**Background:**

The internet has been widely accessible and well accepted by young people; however, there is a limited understanding of the internet usage patterns and characteristics on issues related to health problems. The contents posted on online health communities (OHCs) are valuable resources to learn about youth's health information needs.

**Objective:**

In this study, we concurrently exploited statistical analysis and topic analysis of online health information needs to explore the distribution, impact factors, and topics of interest relevant to Chinese young people.

**Methods:**

We collected 60,478 health-related data sets posted by young people from a well-known Chinese OHC named xywy.com. Descriptive statistical analysis and correlation analysis were applied to find the distribution and influence factors of the information needs of Chinese young people. Furthermore, a general 4-step topic mining strategy was presented for sparse short texts, which included sentence vectorization, dimension reduction, clustering, and keyword generation.

**Results:**

In the Chinese OHC, Chinese young people had a high demand for information in the areas of gynecology and obstetrics, internal medicine, dermatology, plastic surgery, and surgery, and they focused on topics such as treatment, symptoms, causes, pathology, and diet. Females accounted for 69.67% (42,136/60,478) and young adults accounted for 87.44% (52,882/60,478) of all data. Gender, age, and disease type all had a significant effect on young people's information needs and topic preferences (*P*<.001).

**Conclusions:**

We conducted comprehensive analyses to discover the online health information needs of Chinese young people. The research findings are of great practical value to carry out health education and health knowledge dissemination inside and outside of schools according to the interests of youth, enable the innovation of information services in OHCs, and improve the health literacy of young people.

## Introduction

### Background

To live a healthy life, people may pay greater attention to the information related to physical and mental health, disease, nutrition, and health protection. Heath information can guide health and clinical behaviors [1,2], and the availability of the internet makes it convenient to retrieve health-related information [3]. According to the search behavior report on popular science needs of Chinese citizens in 2018 [4], health and medical science rank the first in the search index among the popular science topics concerned, with a search proportion of 66.8%. The large number of users and the convenience of information access make online health communities (OHCs) one of the most important sources for searching and exchanging health-related information, experiences, advice, support, and opinions [5]. The large-scale sharing of health information also makes OHCs a valuable and abundant source of data for addressing public health questions [6]. Therefore, user-generated content in OHCs is one of the most direct and convenient ways of learning the topics of interest for users [7].

Young people are the future and the hope of all nations, thus promoting the health of young people is an important part of the strategy of a healthy China. Youth aged between 10 to 19 years face a range of health risks and this age is an important developmental period when health behaviors, values, and attitudes are established; these are often carried into adulthood [8]. According to the definition of young people from World Health Organization (WHO), we defined those between 10 and 25 years of age as young people, and within this group, those between 18 and 25 years old as young adults and those between 10 and 17 years old as minors, to provide a deeper understanding of the characteristics of health-related internet usage for this important age group [9].

Although the internet is widely accessible and well accepted by young people, there is a limited understanding of internet usage patterns and characteristics on issues related to health problems [10]. Despite the importance, little progress has been made to meet the need of providing online health information. Research on young people’s online health mostly rely on data collected from questionnaire surveys or interviews, with the number of data samples being fewer than 1000 [11-13]. These can hardly be expected to represent the actual information needs of young people. The related data analyses have been mostly based on basic statistics and correlation of questionnaire data and interview data [13-15], and few studies have been performed with the aim of understanding the user-generated content through natural language processing (NLP) techniques to discover the topics and interests of youth.

The analysis of content of online health information, however, is very hard. The user-generated question and answer text data in OHC is often short in length and sparse in content, and the sparsity in short-text documents poses great challenges for topic analysis. Classic topic models such as latent Dirichlet allocation [16] and probabilistic latent semantic analysis [17] fail to work effectively due to the lack of word co-occurrence patterns in each short document [18,19]. Another feasible way to realize topic analysis for short text is based on word embedding models, such as Word2Vec [20]. However, such models usually use static coding methods and only consider the local information of the text. Without the overall information, this method cannot distinguish feature words by context semantics [21]. In addition, because of the sparsity, the feature vector cannot represent the semantics of short text well.

### Related Work

In this section, we summarize the related work that investigated the online health information need of young people, including the work on data collection, data analysis methods, and the discovered topics.

The growth of the internet has made health information more accessible than ever before [22]. For young people, the daily internet access rate is generally high, and the internet has become an important resource to support their self-care and health-related activities and services [10].

Many studies have been made to understand the online health information needs of young people. The data collection approaches used include questionnaire survey, interview, and web crawler collection [23-25]. The corresponding data analysis methods are also different. For the questionnaires and survey data, descriptive statistical analysis, correlation analysis, and multiple logistic regression analysis are generally applied [11,12,14]. For interview data, many studies use content analysis and statistical analysis [13,15]. Recently, with the increase of user-generated content from OHCs, social media, and health service websites, some researchers have begun to collect data through web crawler and to develop text mining techniques, such as topic analysis and sentiment analysis, to discover user health information needs [26-28]. For example, text mining technology was used to analyze the pregnancy data of MedHelp in OHCs, and the adopted and unused answers were classified with a support vector machine–radial basis function kernel classification algorithm [26]. Based on the extracted information of 1000 consultation records from one OHC, the features of the health information needs of patients with hypertension were explored by content analysis and clustering analysis [28].

A variety of studies have been conducted to find the topics of interest of young people from online health information [10]. The results indicate that most online health information is closely related to the self-development of young people. The topics include daily health-related issues [29-31], physical growth [13], mental health [32,33], sexual and reproductive health [34-36], and physiological diseases [34,37]. Daily health-related issues, such as exercise and nutrition, beauty and skin care, fitness and diet, flu, and infection draw significant attention from young people [29]. They also use internet information on symptoms and treatment options for physiological diseases, such as arthritis or diabetes, and may turn to alternative sources according to the topic [34,37]. Young people who experience mental health issues often seek help and information related to their psychosocial health and advice from peers or doctors online [24,34]. For sexual health issues, both males and females are likely to look for information and help about such sensitive topics [24,34]. The internet has become a major resource for young people in supporting their self-care and health-related activities and services. The actual needs of young people may vary across different countries or different age groups [11,12,38,39].

Although many studies have been made on the online health information of young people, the number of samples for most is small and does not adequately reflect the general needs of youth. Moreover, previous studies have generally not been based on user-generated content nor have they used NLP technology to develop further research.

### Objective

To fill the gap of current research, this paper presents a framework with a set of techniques to analyze online health information of interest to youth in China. The main contributions of this paper are the following.

We propose a topic analysis scheme to extract information from short-text messages in 4 steps: sentence vectorization, dimension reduction, clustering, and keyword generation. We used the advanced pretrained Siamese network model sentence-BERT (SBERT) to generate high-quality sentence vectors and principal component analysis (PCA) to reduce the vector dimension for more effective clustering. These techniques can be extended to apply to other topic extraction tasks based on short texts from the internet.

Concurrently exploiting statistical analysis and topic analysis, we also explored the distribution, impact factors, and topics of interest based on the online health information of Chinese young people posted on a popular Chinese OHC. The research findings are of great practical value to carry out health education and health knowledge dissemination inside and outside schools according to the interests of youth, enable the innovation of information services in OHCs, and improve the health literacy of young people.

## Methods

### Study Design

**Figure 1:**
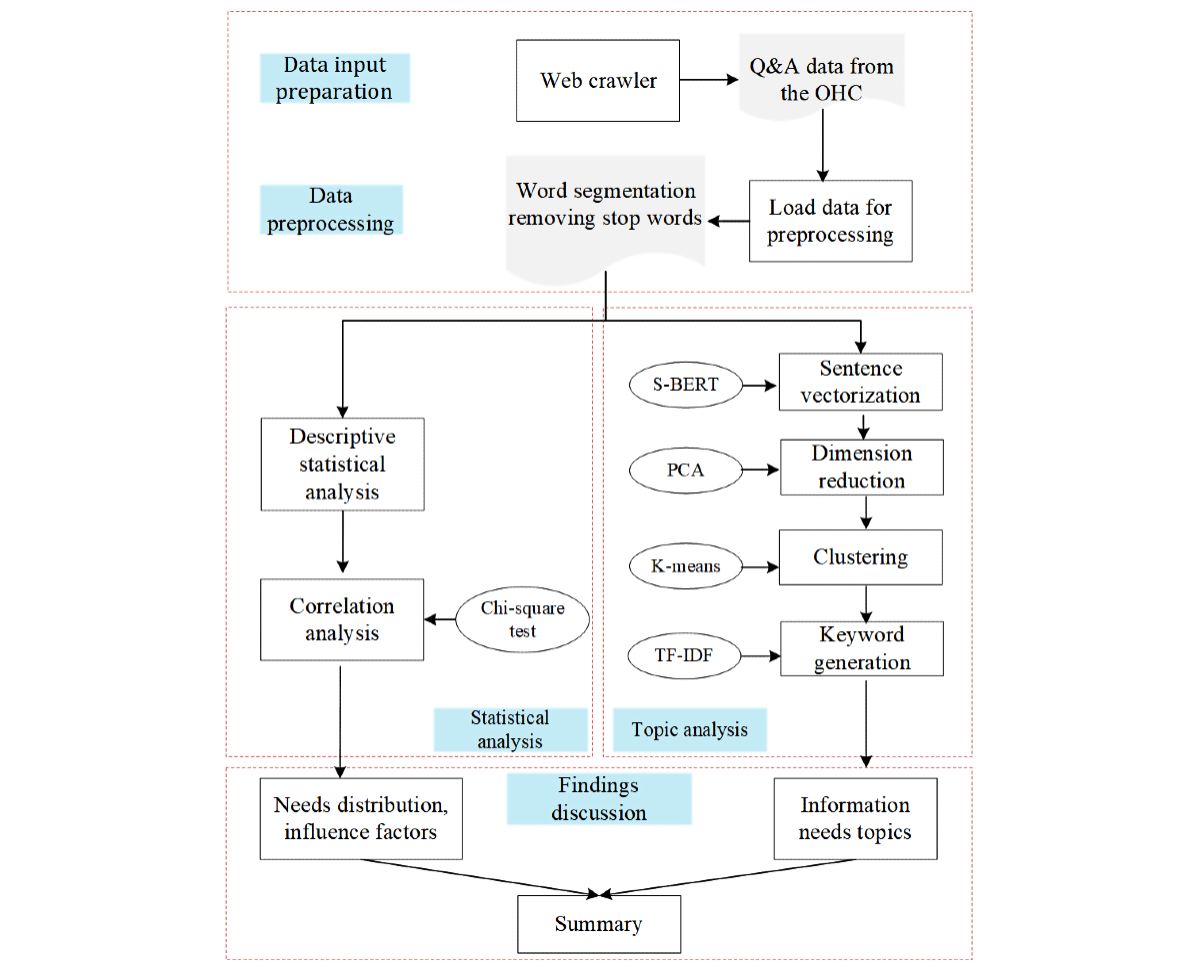
Research framework. OHC: online health community; PCA: principal component analysis; Q&A: question and answer; SBERT: sentence-BERT; TF–IDF: term frequency–inverse document frequency.

The overall research framework is displayed in Figure 1. It was divided into 4 major steps: input data preparation, data preprocessing, data analysis, and findings discussion. Among these, the data analysis consisted of 2 parts: statistical analysis and topic analysis. The statistical analysis part applied descriptive statistical analysis and correlation analysis to find the distribution and major factors related to Chinese youth’s information needs. Meanwhile, the topic analysis part used a 4-step strategy to mine the topics of specific diseases. In the 4-step topic extraction strategy, the first step used the representative pretrained language model SBERT to realize the sentence vectorization. The PCA algorithm was then used to reduce the vector dimension to improve the clustering efficiency and accuracy. After the optimal number of clusters was determined by the silhouette coefficient, a *k*-means clustering algorithm was adopted to get *k* clusters, term frequency–inverse document frequency (TF-IDF) was applied to acquire the keywords of each cluster, and the information needs topics were generated.

### Input Data Preparation

Our data set was collected by a web crawler from a popular Chinese OHC named xywy.com, which allows users to publish health-related questions in many different disease categories. xywy.com is one of the OHCs that had explored and implemented medical and health services in China earlier. As the pioneer of OHC, its completeness and accuracy of information content are widely recognized [40].

A total of 60,478 question and answer messages posted by young users from June 1, 2019, to June 1, 2020, were collected as input data. Each message contained a set of tags, including user gender, age, question time, department affiliation, question title, question content, and doctor’s responses.

### Data Preprocessing

There were 2 important steps in data preprocessing: word segmentation and removing stop words. As the data source in this study was closely related to medical and health terms, the accuracy of word segmentation could be improved by combining them with a Chinese medical thesaurus. In this study, the Jieba library and Chinese medical thesaurus, CMesh [41], were used together to facilitate the word segmentation.

Removing the stop words that convey little useful meaning can reduce the dimension of the feature space [42]. Therefore, after applying the Baidu stop-word table, we removed all stop words, including articles, conjunctions, pronouns, and linking verbs.

### Topic Extraction Strategies

We created a set of questions about a specific disease, *Q*= {*q*_1_, *q*_2_, …*q*_|Q|_}. It contained |Q| questions, and *q_i_* was the *i*-th question in *Q*. For topic extraction from *Q*, we needed to first cluster questions in *Q* into *k* clusters *C_1_, C_2_,* …*C_k_*, and then generate *N* key words to provide the topic of cluster *C_j_*.

#### Sentence Vectorization

To extract topics from *Q*, the first thing was to represent the short question text data *q_i_* in *Q* with an appropriate form to calculate the distance between question texts. As mentioned earlier, standard topic models and general word embedding methods were not suitable for this task. Therefore, we applied an effective pretrained NLP model in this step.

BERT [43] is now widely used in various NLP tasks. However, the sentence representation generated by BERT is not efficient for a clustering purpose. As BERT requires 2 sentences to be entered into the model at the same time for information interaction when calculating semantic similarity, it results in a significant computational overhead, and experiments [44] have shown the results to usually be even worse than those of some word-embeddings models.

**Figure 2:**
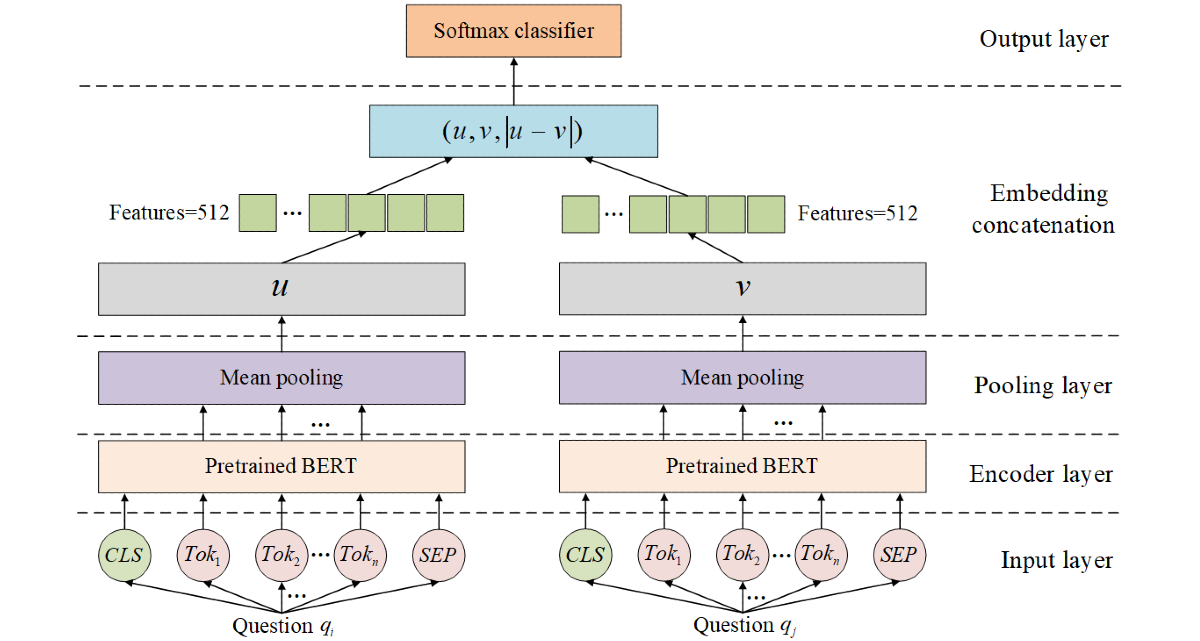
The procedure of sentence vectorization based on sentence-BERT. CLS: a sign placed at the beginning of a sentence for subsequent classification tasks; SEP: a sign placed between 2 sentences to distinguish them; TOK: token embedding.

Instead, we chose the improved pretrained model SBERT [44] to generate sentence vectors for the question text in *Q*. As shown in Figure 2, SBERT used Siamese network structure to generate semantically meaningful sentence vector representations. In the input stage, sentences *q_i_* and *q_j_* were each encoded by pretrained BERT. After that, the 2 sentences were normalized through a pooling layer to obtain the fixed-length vectors *u* and *v*. After this, the (*u, v,* |*u-v*|) concatenated by *u*, *v*, and |*u-v*| was passed through the softmax layer to acquire the classification labels of the 2 sentence vectors, where |*u-v*| denoted the element-wise difference between *u* and *v*. SBERT directly used the cosine similarity to compare the similarity between 2 sentence vectors, which greatly improved the speed of inference while maintaining accuracy.

#### Dimension Reduction for Sentence Vectors

After punctuation and invalid symbols were removed, *q_i_* in *Q* had the average length of 45 Chinese characters. For each *q_i_* in *Q*, SBERT generated a 512-dimension vector. A higher dimension causes more computation overhead and prevents the cluster algorithm from achieving better results on a relatively large input data set. We thus chose to use the PCA technique to reduce the vectors dimension, which is an effective method to process, compress, and extract information based on the covariance matrix of variables.

To reduce the dimension of *n* *m*-dimensional vector matrix *R^n^* ^×^ *^m^* generated by SBERT, we first calculated the eigenvalues and eigenvectors of the correlation matrix *R* of *R^n^* ^×^ *^m^*, and then *R^n^* ^×^ *^m^* was projected to the eigenvector space *R^n^* ^×^ *^k^* corresponding to the first *k*-dominant eigenvalues whose cumulative contribution rate was *λ*. That is, the original vector was reduced from the *m*-dimension to the *k*-dimension.

#### Sentence Vector Clustering

For topic extraction, we first clustered sentence vectors output by SBERT. In this step, it is necessary to measure the distance (or similarity) between 2 sentence vectors and determine the number of clusters to form.

In this study, all the sentence vectors generated by SBERT had the same length, and the cosine distance [45] was used to measure the similarity between 2 sentence vectors. *k*-means clustering algorithm was then adopted to get *k* clusters, with each cluster being a topic for a specific disease.

The clustering number *k* had an important influence on the clustering results of the *k*-means algorithm, and we used the silhouette coefficient [46] to evaluate the clustering effect, which combined 2 factors, cluster intracohesion and cluster interdissimilarity.

#### Generation Of Keywords

Keywords needed to be generated to describe the topics of interest in different clusters. The representation method based on frequent values has often been used because it reduces the text dimension and has a better effect [47]; we thus applied the TF-IDF algorithm [48] to extract keywords from the clusters results.

For each cluster *C_j_*, TF-IDF was used to calculate the importance of words in *C_j_*, and key words were selected based on the importance level of words. After the high word frequency in a cluster and the low text frequency in the disease question set were combined, the top *N* words with high word importance levels were selected to generate the topic words for a certain disease, and *N* was the user setting parameter. Thus, the topics of information needed for a certain disease were generated according to the topic words of each cluster.

## Results

We conducted a set of experiments over the real user-generated data set crawled online to reveal the distribution, influence factors, and topics of interest of Chinese young people.

The models and algorithms in this paper were programmed based on Python 3.6 (Python Software Foundation) under the deep learning framework PyTorch 1.5.1 and TensorFlow 1.14.

To evaluate the clustering effect for short-text based on the sentence vectors generated by BERT and SBERT, we selected 8701 samples from the whole data set that had disease labels from different departments. Experiment results showed that the clustering effect was significantly improved by SBERT, with adjusted Rand index [49], adjusted mutual information [50], and Fowlkes and Mallows index [51] evaluation metric values of 32.1%, 28.6%, and 25.1% higher than those of BERT, respectively.

### The Results of Statistical Analysis

**Figure 3:**
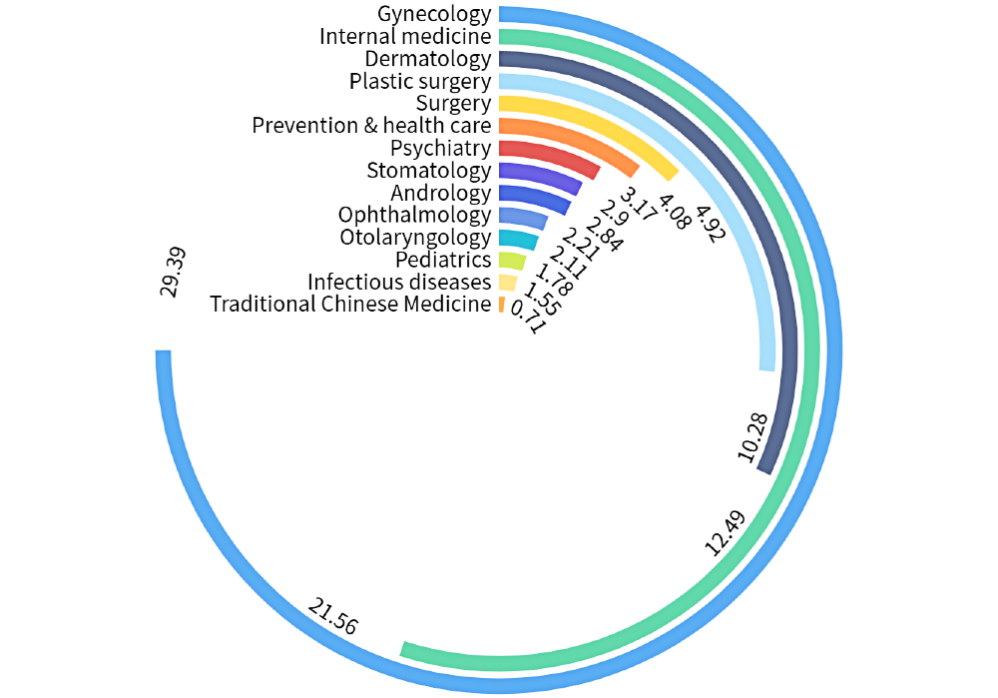
Distribution of Chinese young peoples’ health information needs.

The distribution of Chinese young people's interests based on the collected data of the health information is shown in Figure 3. Based on the percentage of the question data, the needs were mainly concentrated in gynecology and obstetrics, internal medicine, dermatology, plastic surgery, and surgery.

**Figure 4:**
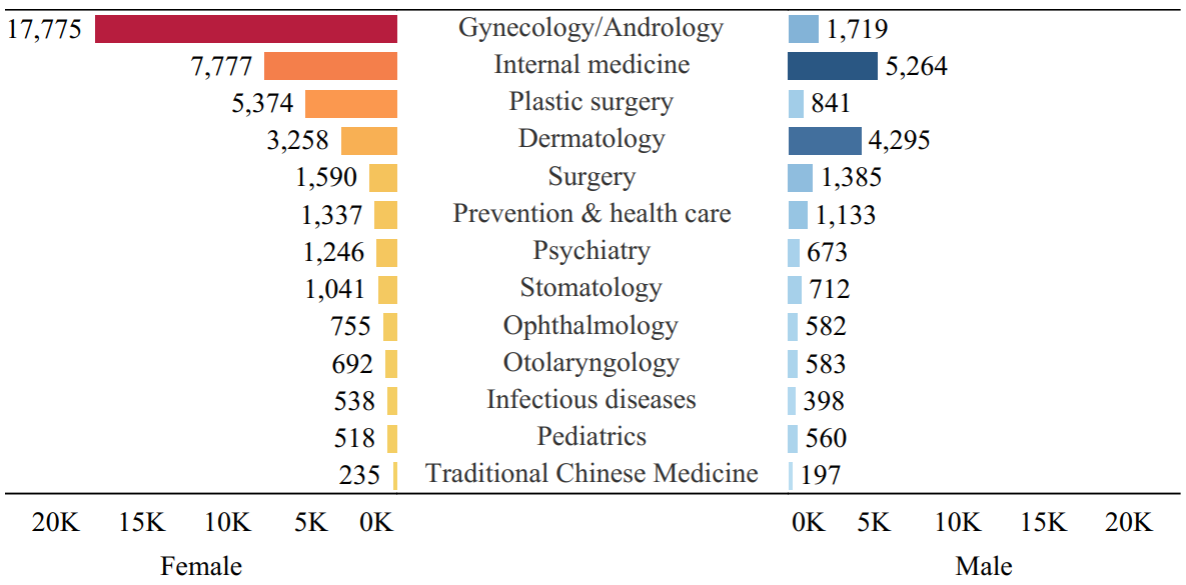
Health information needs distribution of Chinese young people by gender.

Statistical data indicated that the ratio of female to male gender distribution was about 100:116.9 in China [52]; however, the ratio of the number of questions raised by female to male users in our collected data was about 229.72 (n=42,136) to 100 (n=18,342), which showed that the young female users were more willing to use OHC for health consultation than were the male users. The results of the chi-square test between gender and departments showed that there were significant differences in health interest areas between different genders (*X*^2^_1_*=*17004.9; *P*<.001). As shown in Figure 4, the information needs of female users were mainly concentrated on the departments of gynecology and obstetrics, internal medicine, plastic surgery, and dermatology, while those of males were mainly focused on internal medicine, dermatology, andrology, and surgery. It could also be seen that both male and female young people tended to use the OHC to get help about sex-related issues, with females being more concerned about plastic and cosmetic issues and men being more concerned about surgical issues.

In terms of age distribution, the willingness to access health information from the OHC appeared to increase as age increased. Young adults aged 18 to 25 years were the main group of young users in the OHC, accounting for 87.44% (52,882/60,478) of the total number. The results of the chi-square test between age and departments showed that there were significant differences in the health information need areas of young people at different ages (*X*^2^_1_*=*4437.6; *P*<.001). The department distribution of the needs at different ages is shown in Figure S1 in Multimedia Appendix 1. The young adults’ needs were mainly concentrated on gynecology and obstetrics, internal medicine, dermatology, and plastic surgery, which was basically consistent with the overall distribution of needs. The information needs of minors were mainly in the areas of internal medicine, gynecology and obstetrics, dermatology, pediatrics, and preventive health care.

### The Results of Topic Analysis

To further explore the topics of interests of Chinese young people related to health information, we first selected 5 representative diseases, including irregular menstruation, influenza, vitiligo, weight loss, and depression. After applying our 4-step topic extraction strategy, keywords were generated and topics were extracted for each selected disease. The top *N* key words of each cluster ranked by the word importance were selected to characterize the topics. Unless otherwise specified, *N* was set to 10 in this study.

To better understand the experiment results, a table and word cloud were used to display the topic extraction results. The final topic extraction results of menstrual irregularities and weight loss are presented in Table 1 and Table 2, the topics of vitiligo are presented in Table S1 in Multimedia Appendix 1, while the results of influenza and depression respect are shown in the form of word cloud in Figure S2 and Figure S3 in Multimedia Appendix 1, respectively. The keywords here eliminated words such as “vitiligo,” and “ influenza,” and other disease name words, such as well as “how” and “what,” along with other meaningless words.

**Table 1:** Topic extraction results for menstrual irregularities.

Topic	Frequency, n (%) (N=1400)	Concrete content	Keywords (top 5)
Treatment	625 (44.64)	Consult for treatment of menstrual irregularities and medications	Delay, how to treat, how to do, dysmenorrhea, causes
Pathology	84 (6.00)	Consult for types, etiology, and pathology of menstrual irregularities	What is going on, menstruation, bleeding, causes, brown
Symptom	242 (17.29)	Consult for signs, symptoms, and tests of menstrual irregularities	Delay, leucorrhea, examination, symptoms, feelings
Pregnancy	189 (13.50)	Counseling whether menstrual irregularities are associated with pregnancy	Pregnancy, have sexual intercourse, birth control pills, boyfriend, safety period
Diet	260 (18.57)	Consult for menstrual irregularities, dietary contraindications and precautions, and suitability of certain foods	What to eat, food, conditioning, diet, brown sugar

**Table 2:** Topic extraction results for weight loss.

Topic			Frequency, n (%) (N=3381)		Concrete content		Keywords (top 5)
**Diet**			2371 (70.11)				
	Coarse grain	411 (12.16)		Counseling on coarse grain cereals that help lose weight, as well as consumption effects		Potatoes, sweet potatoes, corn, red beans, oats	
	Fruits and vegetables	487 (14.40)		Counseling on fruits and vegetables that help you lose weight and how they work		Apples, fruits, bananas, cucumbers, bitter gourd	
	Beverages	353 (10.44)		Counseling on various types of beverages that help with weight loss and how well they work		Yogurt, honey water, milk, diet tea, coffee	
	Weight loss recipes	1120 (33.13)		Counseling on healthy recipes that help to lose weight		What to eat, how to eat, food, effect, dieting	
Surgery			614 (18.16)		Counseling on various surgical weight loss methods, effects, and costs.		Diet, treatment, liposuction, thin face pin, surgery, lipolysis, effect
Pathology			396 (11.71%)		Counseling on the causes of obesity and weight loss methods		Obesity, getting fat, causing, sweets, why

Overall, the topics for all types of diseases were mainly focused on treatment, symptoms, pathology, and diet. For irregular menstruation, influenza, and vitiligo, young people were most concerned about the topic of treatment. Unlike other diseases for which users were mainly concerned about the treatment method, patients with vitiligo were also concerned about the treatment cost and location of treatment. Young people consulting on weight loss were most concerned about the role of diet in weight loss, including the information on how to choose diet recipes and the types of roughage grains, fruits and vegetables, and beverages that help with weight loss. In contrast to other physiological disorders, young people under the depression department were not concerned about the diet topic. They were more interested in symptoms than in treatment. The results of the chi-square test between the disease type and the information needs topic showed that there were significant differences in the topic of information needs between young people with physical and psychological disorders (*X*^2^_1_*=*2591.7; *P*<.001).

The gender distribution of vitiligo and influenza is shown in Figure S4 in Multimedia Appendix 1. The chi-square tests between young people's gender and information need topics in the data of influenza (*X*^2^_1_*=*113.7; *P*<.001), vitiligo (*X*^2^_1_*=*100.6; *P*<.001), weight loss (*X*^2^_1_*=*49.0; *P*<.001), and depression (*X*^2^_1_*=*88.7; *P*<.001) all indicate that there were significant differences in topics of interest in young people of different gender. In all 4 diseases, male young people asked more questions on the treatment topics and pathology topics than did females, and female young people asked more questions on the diet topics than did males.

**Figure 5:**
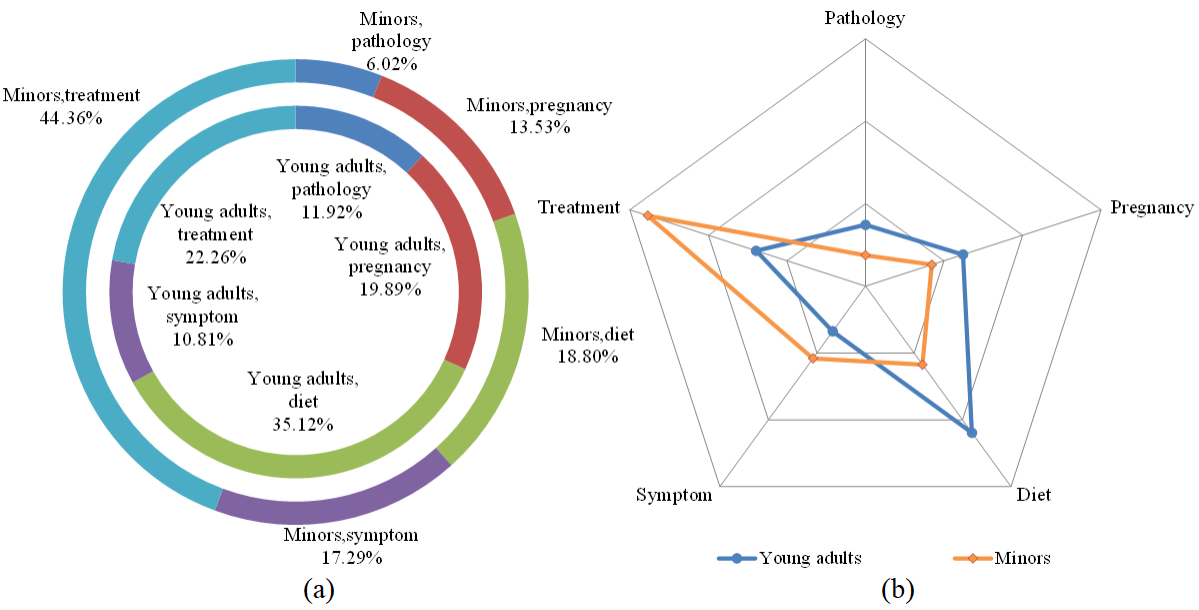
(a) Distribution map of menstrual irregularities topic distribution at different age stages. (b) Radar chart of menstrual irregularities topic distribution at different age stages.

In Figure 5, we used the distribution map and radar chart to show the information needs topic distribution of different ages for menstrual irregularities. The chi-square tests of irregular menstruation (*X*^2^_1_*=*44.4; *P*<.001), influenza (*X*^2^_1_*=*81.1; *P*<.001), vitiligo (*X*^2^_1_*=*64.2; *P*<.001), weight loss (*X*^2^_1_*=*157.5; *P*<.001), and depression (*X*^2^_1_*=*30.2; *P*<.001) indicated that there were significant differences in health information needs of young people of different ages. In all the 5 selected diseases, young adults asked a higher proportion of questions on the topic of diet than did minors, indicating that young adults were more concerned about the topic of diet than were minors.

## Discussion

### Principal Findings

There are four principal findings in this study. First, Chinese young people's interests on online health information are mainly distributed in the following areas in descending order: gynecology and obstetrics, internal medicine, dermatology, plastic surgery, and surgery. It is worth noting that sexual and reproductive health issues are a concern of both Chinese male and female young people. The development of sexual organs and the awakening of sexual consciousness during adolescence likely lead young people to pay greater attention to health issues related to sexual organs and the reproductive system, including sexual organ development, urinary infection, and menstrual irregularities. However. because of the cultural background and relatively poor sex education level in China, young people are often shy to talk about sexual problems offline, so they hope to receive helpful information online [53]. Young people have a high level of oil secretion in their skin, and bad habits and dietary habits are common, which not only affect the health of their skin but also the aesthetics of their appearance, so health information related to skin problems and cosmetic surgery is also urgently sought out by young people.

Second, most young people in Chinese OHCs are female; this is because there are significant gender differences in the level of health awareness and attention to health information among young people in social media [54], with females having a higher level of health awareness and attention to health information than males. Moreover, gender is an important factor affecting the need for health-related information for Chinese young people. Male young people are more concerned about treatment and pathology topics than are females, and female young people are more concerned about diet topics than are males. This is the same conclusion as that found by previous studies [55], where male young people were significantly lower than females both in terms of their level of dietary health and awareness of a healthy diet.

Third, young adults aged 18 to 25 year are the main group of young users in OHCs. This is because the number of young adults using the internet and OHCs is much larger than that of minors aged 10 to 17 years. As a group that has initially left the family and entered society, young adults lack parental care and help in health issues and are more willing to seek help in OHCs. There are also significant age differences in young people's health information needs in OHCs. Compared with young adults, the interest in gynecology and obstetrics is lower while the interest in pediatrics is higher among minors, and this difference is mainly determined by the developmental stage. Furthermore, young adults are more concerned with the topic of diet than are minors. This is because primary and secondary schools in China do not currently provided adequate dietary health education [56], but as young people grow older, the channels for dietary health education expand, their knowledge of dietary health increases, and their awareness of the importance of healthy eating rises.

Finally, for Chinese young people, the information needs mainly focus on treatment, symptoms, etiology, pathology, and diet, whereas less attention is paid to the topic of prevention. Meanwhile, there are significant differences for different disease types. For physiological diseases, such as irregular menstruation, influenza, and vitiligo, young people pay most attention in OHCs to the treatment to understand the treatment methods, costs, and hospital-related information. For mental diseases, such as depression, they are most concerned about the topic of symptoms, hoping the OHC can help them to judge whether they have the condition or not. This is because young people lack knowledge about psychological health and have difficulty in self-judging mental illness, so more young people are eager to seek help from doctors by describing their symptoms in OHCs to determine whether they have a psychological illness [57].

### Theoretical Implications

Based on real user-generated content, this study applied a web crawler, NLP, and statistical analysis technologies to comprehensively analyze Chinese youth's online information needs. This study attempted to reduce the deficiencies of the related literature, whose limitations included small research samples and relatively simple data analysis methods.

To deal with the challenge of mining topics from a massive collection of sparse short text from the internet, we used a general 4-step topic mining strategy. Using an advanced pretraining model, SBERT, and PCA dimension reduction, we generated high-quality clusters for extracting the topic of health information needs. From a technical point of view, this scheme provides a good method of topic analysis for short texts collected from the internet. Furthermore, with minor changes, such as removing word segmentation in the data preprocessing step, it can be extended to apply to other similar tasks using English-language data from websites.

Our study also found that there were significant differences between Chinese and other countries' youth in the distribution and topics of online health information needs, which may have important implications for other researchers by providing data support and a basis for further research on differentiation.

### Practical Implications

Many practical implications could be derived from this study. First, the education of disease prevention for young people should be strengthened. The topic mining results of various diseases showed that youth pay the least attention to the topic of disease prevention, which indicates that schools, families, and internet health and service platforms including OHCs should pay more attention to the education and guidance of disease prevention for youth.

As mentioned earlier, sexual and reproductive health were one of the most concerning fields for Chinese young people. Therefore, it is necessary to improve network management to guide youth in treating and understanding sex-related information on the internet. An effective way is to establish professional and authoritative sexual health–related knowledge platforms to provide scientific information to young people at different stages of development.

Moreover, the information service mode of OHCs requires innovation. At present, most of the information service models of Chinese OHCs are centered on the aggregation and organization of health information resources, which ignores the needs of users to some extent and is challenged in providing accurate service. Therefore, databases on user's health information needs should be established based on the results from mining and analysis of their actual interests. Based on the OHCs’ service platform, information matching and precision service of the information resources and information needs databases should be realized, which will provide personalized information and health services for youth and other users.

### Limitations

This study has some limitations. First, the experimental data were collected from only a single website, and thus the data source setting was substantially limited. In future studies, we plan to collect a larger data set from different OHCs to ensure the research results are more comprehensive and reliable. Second, although the presented framework showed good results in topic mining tasks for short texts from the internet, there is still much room for improvement related to the clustering tasks in specialized domains, and our future work will integrate expertise in specific domains into the model to improve its performance.

### Conclusions

In this study, we conducted statistical analysis and topic analysis of online health information to explore the distribution, impact factors, and topics of interests of Chinese young people. A general topic analysis strategy using the pretraining model SBERT was proposed to extract high-quality topics based on large-scale sparse short texts from the internet. The research findings are helpful for health education departments to understand the real health-related needs of young people, carry out targeted education, and improve young people’s health literacy, and may be useful for OHCs to innovate and improve information service.

## Appendix

Multimedia Appendix 1 Related work.

## Abbreviations

OHC: online health community
NLP: natural language processing
PCA: principal component analysis
TF-IDF: term frequency–inverse document frequency
SBERT: sentence-BERT
WHO: World Health Organization
