# Supplementary material for: Health Information Needs of Young Chinese People Based on an Online Health Community: Topic and Statistical Analysis
Source: JMIR Med Inform. 2021 Nov 8;9(11):e30356. doi: 10.2196/30356 (PMC8663605; doi:10.2196/30356)
Supplement: Multimedia Appendix 1 [file medinform_v9i11e30356_app1.docx]

**Multimedia Appendix 1**

Figure S1. The distribution of health information needs of Chinese young people by age.


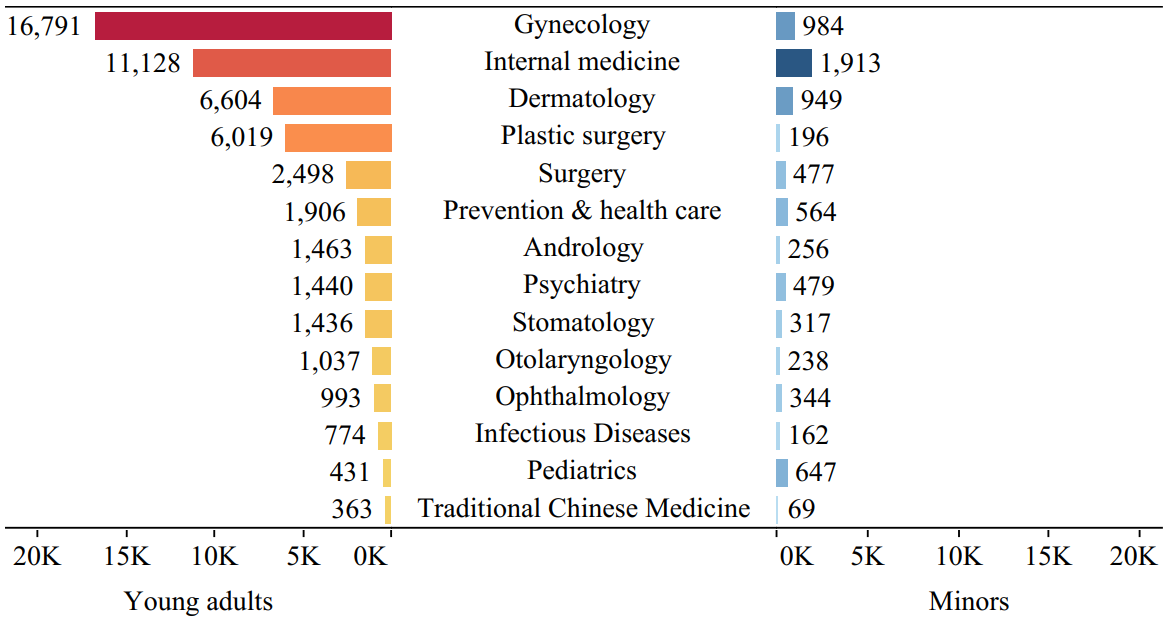


Figure S2. Word clouds (Top 30) in each topic of influenza:(a) The word cloud of symptom topic; (b)The word cloud of pathology topic;(c) The word cloud of dietary prevention topic;(d) The word cloud of Medical treatment topic;(e) The word cloud of diet treatment topic.


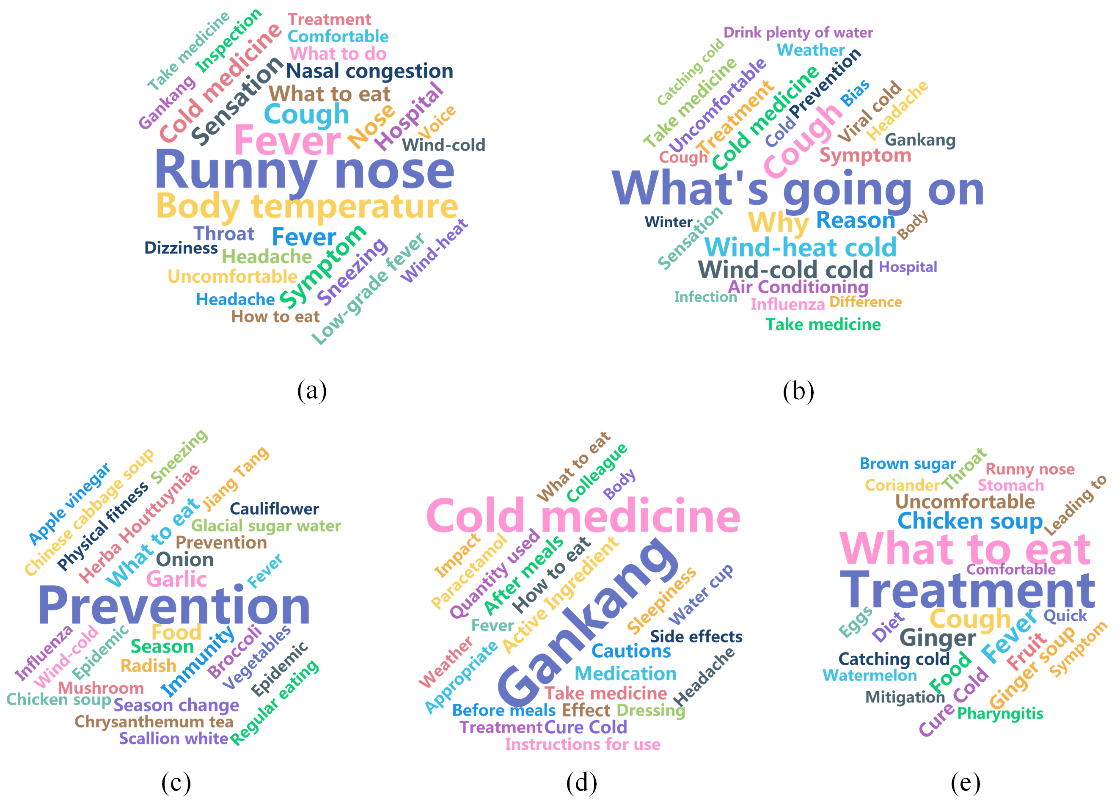


Figure S3. Word clouds (Top 30) in each topic of depression:(a) The word cloud of symptom topic; (b)The word cloud of pathology topic;(c) The word cloud of non-drug treatment topic;(d) The word cloud of drug treatment topic.


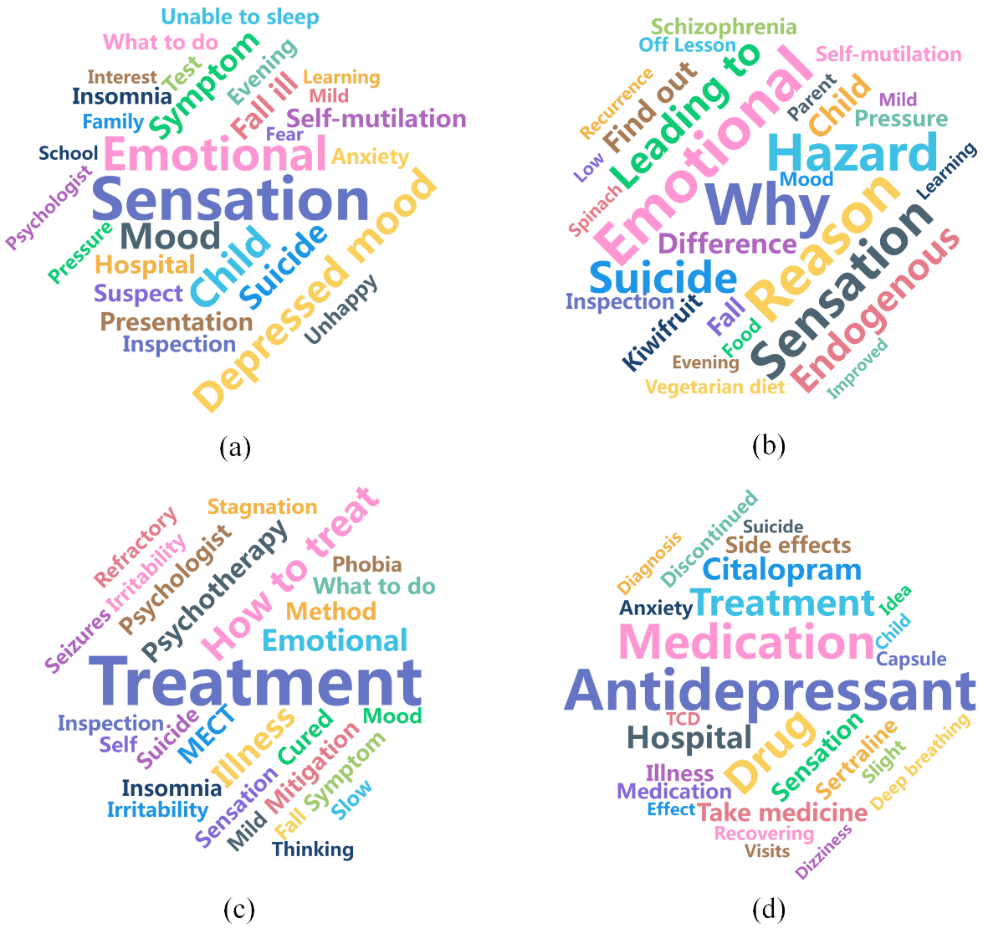


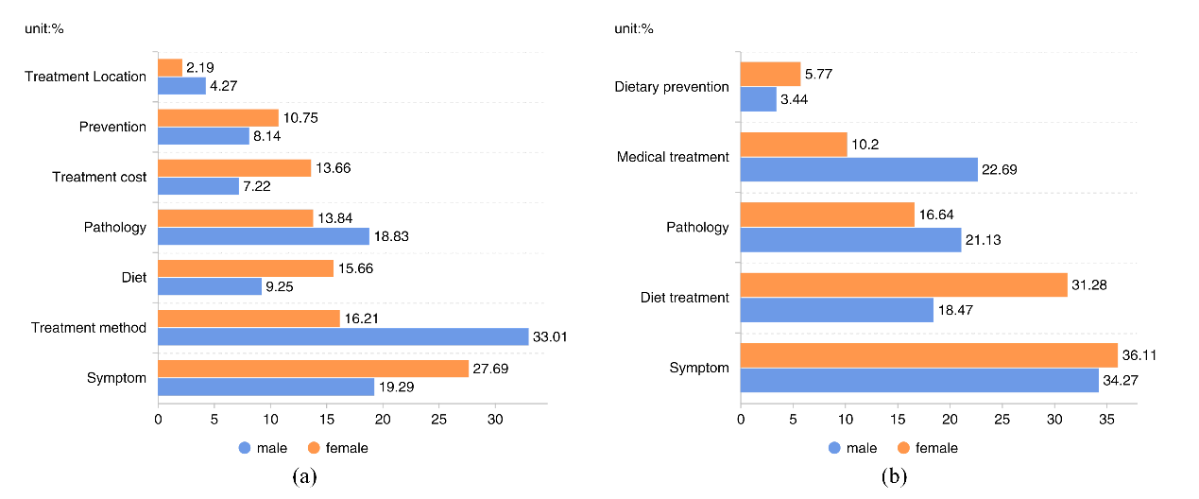
Figure S4. Need topic distribution by genders of Vitiligo and Influenza:(a) Vitiligo’s need topic distribution by genders;(b) Influenza’s need topic distribution by genders.

Table S1. Topics extraction result of Vitiligo.

| Topic | | Frequency, n (%) (N= [2073]) | Concrete content | Keywords (top 5) |
| --- | --- | --- | --- | --- |
| **Treatment** | | 854(41.20%) |  | |
|  | Treatment cost | 185(8.92%) | Consultation for treatment of vitiligo costs, drug prices, etc. | Treatment, fee, how expensive, expenses, spend money |
|  | Treatment method | 592(28.56%) | Consultation for treatment and recovery methods of vitiligo, therapeutic drugs, etc. | Treatment, how to treat, method, cure, effect |
|  | Treatment Location | 77(3.71%) | Consultation for the most authoritative, professional, or best hospital and medical institution for the treatment of vitiligo. | Treatment, hospital, professional, regular, specialized hospital |
| Prevention | | 183(8.83%) | Counseling to prevent vitiligo recurrence, prevent vitiligo spread, seasonal prevention and other issues. | Prevention, method, spread, recurrence, season |
| Diet | | 227(10.95%) | Consultation for dietary regimen for vitiligo, dietary contraindications and precautions, and whether certain foods are suitable for consumption. | Diet, treatment, contraindications, foods, abstain from certain foods |
| Pathology | | 363(17.51%) | Counseling on the type, etiology, and principle of vitiligo. | Symptoms, harms, early, infections, causes |
| Symptom | | 446(21.51%) | Counseling on a part of the body with leukoplakia for vitiligo, disease manifestations and symptoms. | Leukoplakia, body, findings, white spots, symptoms |
